# Steroid-sparing effect and toxicity of dapsone treatment in giant cell arteritis: A single-center, retrospective study of 70 patients

**DOI:** 10.1097/MD.0000000000004974

**Published:** 2016-10-21

**Authors:** Kim Heang Ly, François Dalmay, Guillaume Gondran, Sylvain Palat, Holy Bezanahary, Anne Cypierre, Anne-Laure Fauchais, Eric Liozon

**Affiliations:** a Department of Internal Medicine, University Hospital of Limoges; b Functional unit of Clinical Research and Biostatistics, Limoges School of Medicine, Limoges Cedex, France.

**Keywords:** dapsone, giant cell arteritis, steroid-sparing agent

## Abstract

Although a glucocorticoid (GC)-sparing strategy is needed for patients with giant cell arteritis (GCA) suffering from refractory disease or serious treatment-related complications, evidence of efficacy in this setting of immunosuppressive drugs and biotherapies is lacking. Herein, we evaluated the GC-sparing effects and tolerability of addition of dapsone (DDS) to prednisone therapy in patients with GCA. We retrospectively assessed data on 18 GCA patients who received DDS as a first-line treatment (DDS-1 group) and 52 patients who received it as a second- or third-line treatment for refractory GCA, with or without excessive GC-related toxicity (DDS-2 group). Of these 70 patients, 63 belonged to an inception cohort of 478 patients, whereas the remaining 7 were referred to our department for resistant GCA. In all, 52 patients were assessable for DDS efficacy. The baseline characteristics of the DDS-1 patients were similar to those of 395 GCA patients (control group) who received prednisone alone. DDS-1 patients had a more sustained decrease in GC dose with a lower mean prednisone dose at 12 months, and they comprised higher proportions who achieved GC withdrawal within the first year, who stopped prednisone treatment, and who recovered from GCA (*P* < 0.001 for each variable). Patients in the DDS-2 group achieved a mean rate of prednisone reduction of 65% and a prednisone dose reduction of 16.9 ± 13.3 mg/d. The monthly decreases in the prednisone dose were 2.4 and 1.25 mg in DDS-1 and DDS-2 patients, respectively. DDS-induced side effects were recorded in 44 (64%) assessable patients. These side effects led to lowering of the DDS dose by 25 mg/d in 11 (16%) patients and permanent cessation of DDS in 14 patients (20%), due to allergic skin rash in 7, agranulocytosis in 2, icteric hepatitis in 2, and excessive hemolysis in 2 patients. DDS is a potent GC-sparing agent in GCA that should be evaluated in prospective studies. However, DDS use should be restricted to refractory GCA patients due to its toxicity, and close clinical and laboratory monitoring for 3 months is necessary.

## 1 Introduction

Corticosteroid (CS) therapy is the cornerstone of treatment for giant cell arteritis (GCA), but a treatment course of 18 months to 3 years is usually required. Treatment with CS dramatically alters the symptoms and course of GCA, reducing the likelihood of development of blindness.^[1]^ However, relapses or recurrences frequently occur when CS dosages are tapered,^[2–5]^ resulting in frequent retreatment, CS dependence, and toxicity. Approximately 80% of patients with GCA will eventually experience at least 1 adverse event attributable to CS, and ∼60% will have 2 or more adverse events.^[6–9]^

Adjunctive treatments are needed to reduce the dose and duration of CS therapy and provide longer lasting remission of GCA. Methotrexate (MTX) plays at best a modest role in reducing the relapse rate and lowering the cumulative dose of glucocorticoid (GC) therapy.^[10]^ Addition of infliximab to glucocorticosteroids does not reduce the risk of relapse compared with GC monotherapy.^[11]^ Tocilizumab (TCZ) is effective for induction and maintenance of remission in GC in large vessel vasculitides,^[12–14]^ although its efficacy as a second-line treatment in relapsing patients and the rate of relapse within months after its discontinuation remain to be determined.^[15]^

Dapsone (4–4′diamino-diphenyl-sulfone, DDS), a well known antileprosy drug, has anti-inflammatory effects.^[16,17]^ It is effective against several dermatologic diseases^[18,19]^ and relapsing polychondritis.^[20]^ It has also been occasionally reported as a potent steroid-sparing agent in GCA, with the ability to reduce the cumulative prednisone dose and the total duration of treatment.^[21–23]^ The steroid-sparing effect of DDS could be explained by several mechanisms, including oxygen-radical scavenging, reduction of tumor necrosis factor (TNF)-α and interleukin-8 micro RNA levels, and dysregulation of lymphocyte function in healthy volunteers.^[17]^ In 1986, our department conducted a multicenter, nationwide, randomized study comparing prednisone alone to prednisone plus DDS in patients with newly diagnosed GCA. However, the study was interrupted in November 1987 due to occurrence of DDS-induced agranulocytosis in 2 of 24 patients in the DDS arm. Despite the small sample size (47 patients in total), which hampered identification of a clear-cut steroid-sparing effect, the efficacy of DDS was suggested by a lower rate of GCA relapses, more frequent remission, and a trend toward a shorter prednisone duration, compared with the prednisone-alone group.^[24]^

Although DDS has a relatively poor safety profile with a number of serious untoward effects, such as agranulocytosis, rash, and neuropathy, occurring soon after its initiation,^[25]^ we use it to treat GCA but restrict its use to difficult-to-treat patients. Since no study to date has reported the efficacy and toxicity of DDS as a first- or second-line adjunctive treatment for GCA, we report our experience with DDS use in GCA treatment over a 37-year period.

## 2 Methods

### 2.1 Patients

From January 1976 to February 2016, all patients newly diagnosed with GCA were included in an inception cohort. Before 1990, only patients with biopsy-proven GCA were included in our cohort.^[26]^ After 1990, GCA was diagnosed according to the American College of Rheumatology (ACR) criteria for GCA.^[27]^ Patients with a negative temporal artery biopsy satisfying only 2 ACR criteria (age >50 years and erythrocyte sedimentation rate >50 mm/h) were regarded as having GCA if they also had a vascular PET scan highly suggestive of large vessel GCA.^[28]^ All patients provided written informed consent before participating in this study. Clinical, laboratory, and pathological characteristics were prospectively recorded for 478 consecutive patients, including 351 biopsy-proven patients, using a specific questionnaire that comprised a detailed history and 174 fixed items.^[1]^ Of these patients, 464 (338 biopsy-proven) were recruited since 1979, when DDS was first regarded as a potential steroid-sparing agent for GCA. Before November 1987, DDS was used either as a first-line therapy or in patients who needed to continue prednisone for long periods at a moderate-to-high dose or who experienced serious side effects. A cooperative prospective study was initiated in June 1986 by the French National Society of Internal Medicine (FNSIM) after a preliminary study showed that DDS in addition to corticosteroids had a favorable effect on GCA.^[24]^ In November 1987, the study was interrupted due to a high rate of hematologic complications, including DDS-induced agranulocytosis in 2 patients, and thus we ceased its clinical development. Several years later, DDS was again favored for GCA treatment because of the lack of available efficient GC-sparing agents. Its use was restricted to difficult-to-treat disease, including refractory GCA and patients with serious GC-induced side effects.

Pretreatment clinical, laboratory, and pathological data were recorded prospectively by a senior internist at the time of first admittance to our department. The questionnaire was completed in 96% of the cases. Data from patients recruited since 1990 were stored in real time in a computer file and regularly updated by one of the authors (EL). Individual questionnaires from 87 patients recruited before 1990 were initially stored in a personal library and then transferred to the computer file.

### 2.2 Clinical definitions

Clinical data were defined as reported previously.^[1]^ The temporal arteries were considered abnormal on examination if there were decreased or absent pulses, nodules, redness, thickening, or tenderness in at least 1 artery. Constitutional syndrome was defined by a temperature ≥38 °C for at least 1 week, severe asthenia, and/or weight loss >5%. Jaw claudication was considered present if the patient reported recurring pain upon chewing, which resolved after chewing was stopped, or trismus. Polymyalgia rheumatica (PMR) and nonerosive, seronegative peripheral arthritis are on the rheumatic spectrum of GCA. PMR is defined by at least 2 weeks of moderate-to-severe pain and morning stiffness lasting more than 30 min in at least 2 of the following areas: neck, shoulders, and pelvic girdle. Upper limb artery involvement was defined on a clinical basis (bruits over axillary/humeral arteries, decreased radial pulses, and ischemic arm pain) with confirmation on Doppler-echography studies and/or angiography. Inflammatory markers, blood counts, and hepatic tests were recorded only in GC-naïve patients. Comorbid conditions included hypertension, clinically relevant atherosclerosis (stroke, ischemic heart disease, and lower limb peripheral artery disease), diabetes mellitus, complicated osteoporosis, and psychiatric disorders. Relapse consisted of reoccurrence of clinical symptoms and/or inflammatory parameters, attributed to the GCA, which required increased therapeutics. Such events following planned treatment withdrawal defined recurrence. We also prospectively recorded the occurrence of steroid-induced complications, such as unstable type II diabetes mellitus, severe psychiatric disorders, repeated infections, Cushing habitus, myopathy, and severe osteoporosis with fractures.

No consensual definition of recovery (or prolonged complete remission) in GCA exists. We derived a personal definition to at least 9 months from our experience with the inception cohort. Of 86 registered first relapses, 75 (87%) occurred within the first 7 months. Thus, in our practice, the probability that a patient off steroid treatment will experience a recurrence after this delay is low. Raising the threshold to 12 months would have only resulted in a 3% increase (1 case) in the proportion of patients not truly recovered.

### 2.3 Treatments and monitoring

The majority of patients were treated using standardized GC protocols. Prednisone was administered at 0.6 to 1 mg/kg according to clinical severity. Patients without ischemic manifestations received prednisone 0.6 to 0.8 mg/kg/d until becoming symptom-free and achieving a reduced C-reactive protein level of less than 5 mg/L. Then, the prednisone dose was planned for tapering to 0.35 mg/kg within 4 to 6 weeks. Patients with ischemic manifestations or a threat to their vision (transient ischemic symptoms, abnormal fundus, or abnormal ophthalmic artery flow on Doppler studies) initially received prednisone 0.9 to 1 mg/kg, often preceded by pulse methylprednisolone (daily doses of 300 mg to 1 g, for 1–3 consecutive days), which was thereafter tapered down as mentioned above. Data on DDS treatment were retrieved from the computerized file and individual clinical charts, as needed, and from the FNSIM prospective study database. All patients who received DDS were included, irrespective of treatment duration. These patients were categorized into 2 groups according to the timing of DDS initiation: “first-line” patients (DDS-1) and patients with “difficult-to-treat disease” (DDS-2) for which DDS was considered in addition to prednisone after at least 2 flares or relapses.

### 2.4 Particular caution concerning DDS

We restricted the use of DDS to refractory GCA patients, both experiencing significant CS-related side effects and a protracted prednisone course, with high cumulated doses. As regards TCZ, we used this drug as a third-line treatment (after failure of a MTX or DDS trial) for the aforementioned reasons and since this drug as not yet been approved by the French *Agence Nationale de Sécurité du Médicament*. Before prescribing DDS to a GCA patient, we thoroughly weigh the pros and the cons in a staff consensus and, once the DDS is started, we ensure a close medical supervision of the patient. Strict monitoring was undertaken in every patient, including a complete blood count twice weekly for the first 6 weeks, weekly until week 12, and monthly thereafter. Measurements of the methemoglobin level were performed at the time of DDS initiation, after 1 week, and monthly thereafter. Any reported or observed (on faxed results of blood tests) incident resulted in a quick appointment or the patient's hospitalization, according to perceived severity. Even if the incident seemed minor, we asked the patient to tell us if it is his/her agreement to pursue the DDS treatment and never hesitated to discontinue it in his/her request. This stringent policy may partly explain the high rate of reported DDS-related side effects or discontinuation. We always began the DDS at 50 mg/d with progressive increments up to 100 mg/d, only if necessary and the treatment was well tolerated.

### 2.5 Statistical analyses

Continuous variables were expressed as means ± SD and categorical variables as frequencies and percentages. The normality of the distribution of quantitative variables was checked using the Shapiro–Wilk method. Comparisons of continuous variables were performed using the Mann–Whitney unmatched-pair test. Proportions were analyzed using the chi-squared or Fisher exact test. The significance threshold for all statistical analyses was 0.05. Calculations were performed using the statistical package SAS, release 9.1.3 (SAS Institute, Cary, NC).

Calculation of the mean monthly decrease in the prednisone dose during DDS treatment was obtained by dividing, for each patient, the delta prednisone dose (number of mg/d before DDS introduction and at the time of DDS discontinuation) by the duration (months) of DDS treatment. Mean values were then calculated for DDS-1 and DDS-2 groups as well as the entire series.

## 3 Results

### 3.1 Main characteristics of the series

**Figure 1:**
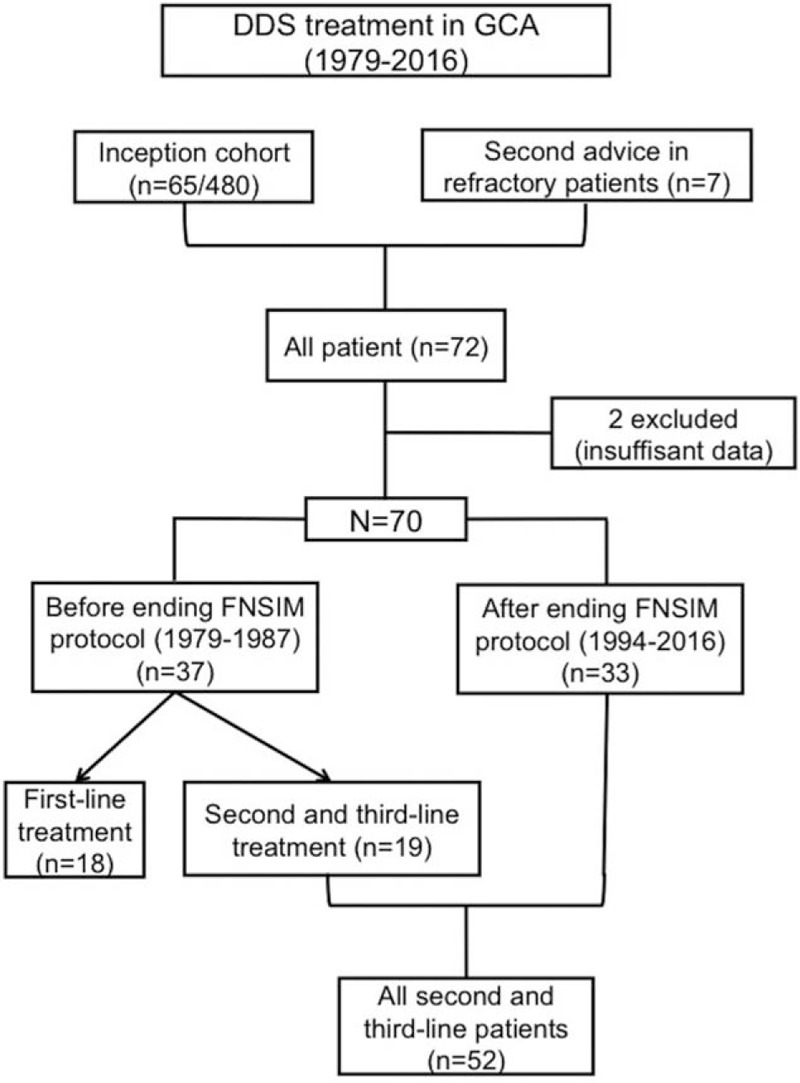
Flowchart for giant cell arteritis (GCA) patients treated by dapsone (DDS). The multicenter prospective trial of DDS as first-line therapy in GCA^[24]^ enrolled 24 patients in the DDS arm and 23 patients in the control arm. All the patients were informed and consented to receive DDS as first- or second-line therapy. Of the 24 Pred-DDS patients, 8 have been included by our department and belong to the present series of 18 patients having received DDS as a first-line treatment (10 patients were treated so before the trial). Data from this trial only reported a 1-year follow-up. We therefore intended to detail a longer follow-up and the final outcomes of these 18 early patients treated with DDS as first-line therapy.

Of the 74 patients intended to receive DDS, 2 declined treatment; thus, 72 patients were treated with DDS in addition to prednisone. Two patients were excluded because of incomplete reports. Of the 70 patients included in the study, 63 belonged to the inception cohort (14%), and the remaining 7 received DDS after being referred to us for difficult-to-treat GCA, most often GC-dependence without major side effects. In total, 18 patients received DDS-1, all of whom were diagnosed before November 1987, whereas 52 patients (DDS-2) had difficult-to-treat disease (29 patients with relapsing or refractory disease, 5 with GC toxicity, and 18 with both) for which DDS was prescribed as a second- (49 patients) or third-line (3 patients) treatment (Fig. 1). The patient's mean age was 72.2 ± 7.7 years (range 56–88 years), and 47 (67%) were females. Before November 1987, for example, the date of multicenter protocol interruption, we used DDS as a GC-sparing agent, as either a first- or second-line treatment, for both complicated and uncomplicated GCA in 37 of 78 (47%) consecutively diagnosed patients, including all 18 first-line treatment patients. From 1988 to January 2016, DDS was prescribed more cautiously, for example, for 26 of 395 (6.5%) patients in the inception cohort and 7 additional patients, all of whom had difficult-to-treat disease. Most patients received DDS in this indication after January 2000. The baseline characteristics of all patients, DDS-1, and DDS-2 patients are shown in Table 1.

**Table 1:**
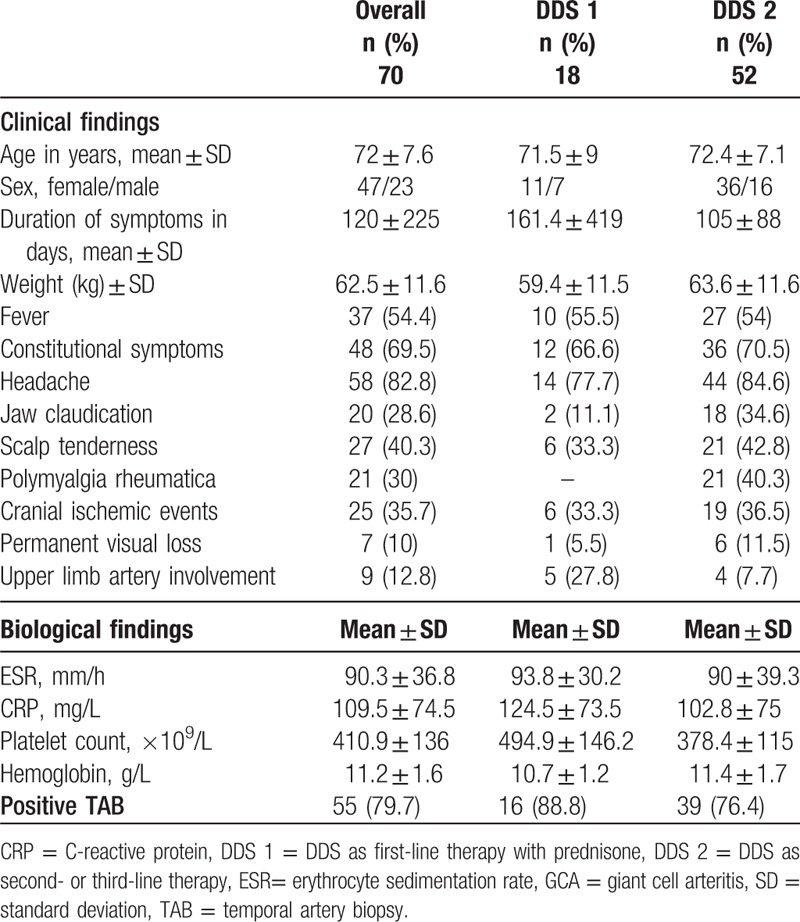
Characteristics of GCA patients treated with DDS.

### 3.2 DDS treatment: timing of initiation, initial doses, and dose adjustments

The delay in DDS treatment initiation ranged from 3 to 54 months (mean 15 ± 12.3 months) after GCA diagnosis in DDS-2 patients: within 6 months in 11 patients, 6 to 12 months in 18 patients, 12 to 24 months in 13 patients, and >2 years in 9 patients. In the FNSIM study (8 patients), the planned initial dose of DDS was 75 mg/d. The mean daily doses of DDS were higher in patients treated before November 1987 (78 ± 16 mg/d) than in those treated later (59 ± 16 mg/d), and the proportion of patients receiving a daily dose ≥75 mg was also higher (92% vs 30%). The mean initial DDS dose was 1.1 mg/kg (range 0.55–2.4 mg/kg). We performed DDS dose adjustment (usually ± 25 mg/d) in 22 patients guided by a clinical response or side effects: increased posology in 8 patients and decreased posology in 15 patients. The mean duration of DDS treatment was 10.5 ± 9.8 months (range 0.5–43 months); it was at least 6 months in 44 patients and 12 months in 28 patients (Table 2).

**Table 2:**
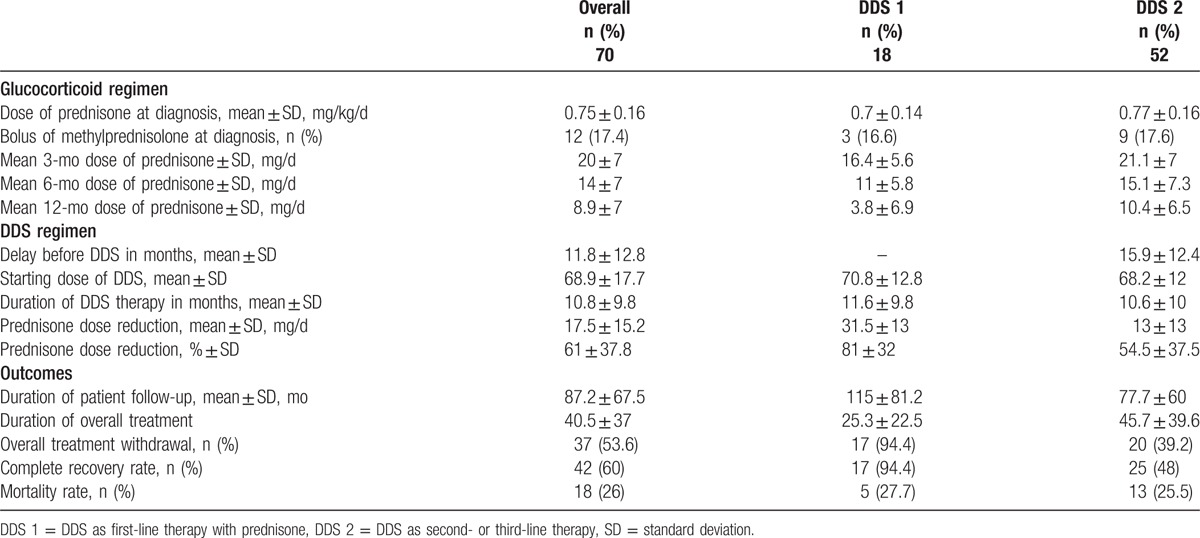
Glucocorticoid and DDS regimens and outcomes in 70 GCA patients treated with DDS.

### 3.3 Glucocorticoid regimens and steroid-sparing effect of DDS

**Figure 2:**
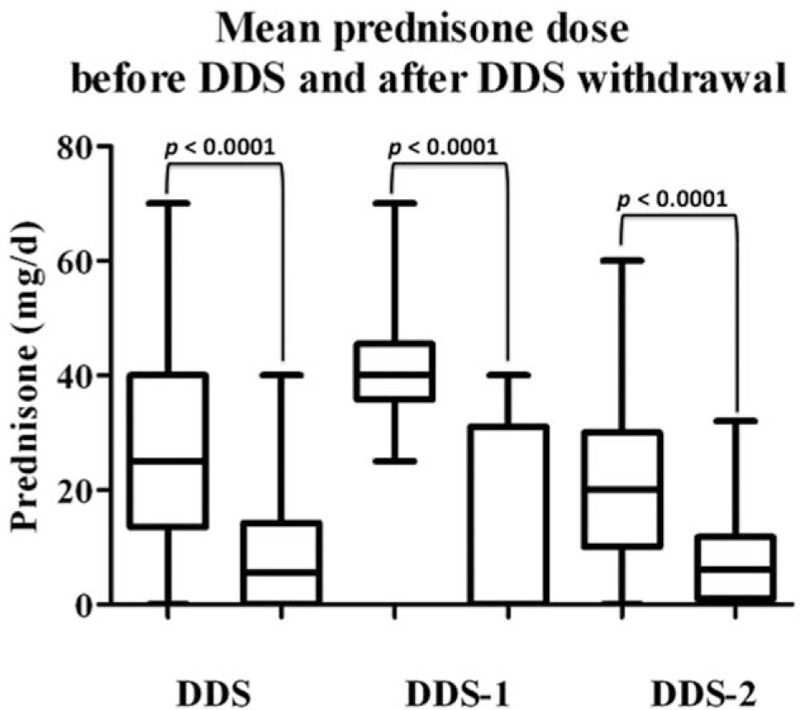
Prednisone dose (mg/d) before dapsone (DDS) and after DDS withdrawal.

In all, 52 patients (14 DDS-1 and 38 DDS-2 patients) were assessable for DDS efficacy (Table 2). These patients received DDS for 13.6 ± 9.7 months (DDS-1, 14.5 ± 6 months; DDS-2, 13.4 ± 10 months), with a significant mean reduction in the prednisone dose in all groups (Fig. 2). Of 15 assessable DDS-1 patients, 8 (53%) had stopped GC treatment within 1 year, 2 of whom relapsed and resumed prednisone for 7 to 10 months. Of these 2 patients, 1 discontinued DDS early due to side effects, while the other took DDS continuously for 27 months and did not relapse thereafter. The mean monthly decrease in the prednisone dose during DDS treatment was 1.5 mg overall, with decreases of 2.4 and 1.25 mg in assessable DDS-1 and DDS-2 patients, respectively.

To assess the steroid-sparing effect of DDS as a first-line adjunctive therapy, we compared the outcomes of DDS-1 patients with those of 395 DDS-naïve GCA patients (control group) included in the inception cohort during the study period (Table 3). The main features at GCA onset were similar between the 2 groups, with the exception of lower frequencies of jaw claudication and polymyalgia symptoms and a lower mean hemoglobin level in the DDS-1 group. The mean prednisone dose at 12 months was lower, and the proportion of patients who discontinued prednisone within 1 year was higher (*P* < 0.0001 for both variables) in the DDS-1 group, although the mean GC treatment duration was similar between the 2 groups. Finally, all DDS-1 patients stopped GC treatment at least once, compared with 59% of DDS-naïve patients (*P* < 0.001). DDS-1 patients achieved a higher rate of recovery from GCA (94% vs 49%, *P* < 0.001), although they flared and relapsed as often as did the control patients. The mean follow-up was longer in the DDS-1 group than in the control group (114 vs 63 months). The total treatment duration for patients who achieved recovery was slightly shorter in the DDS-1 group (14 patients) than in the control group (189 patients): 22.3 ± 16.7 months versus 26.6 ± 16 months (*P* = 0.15).

**Table 3:**
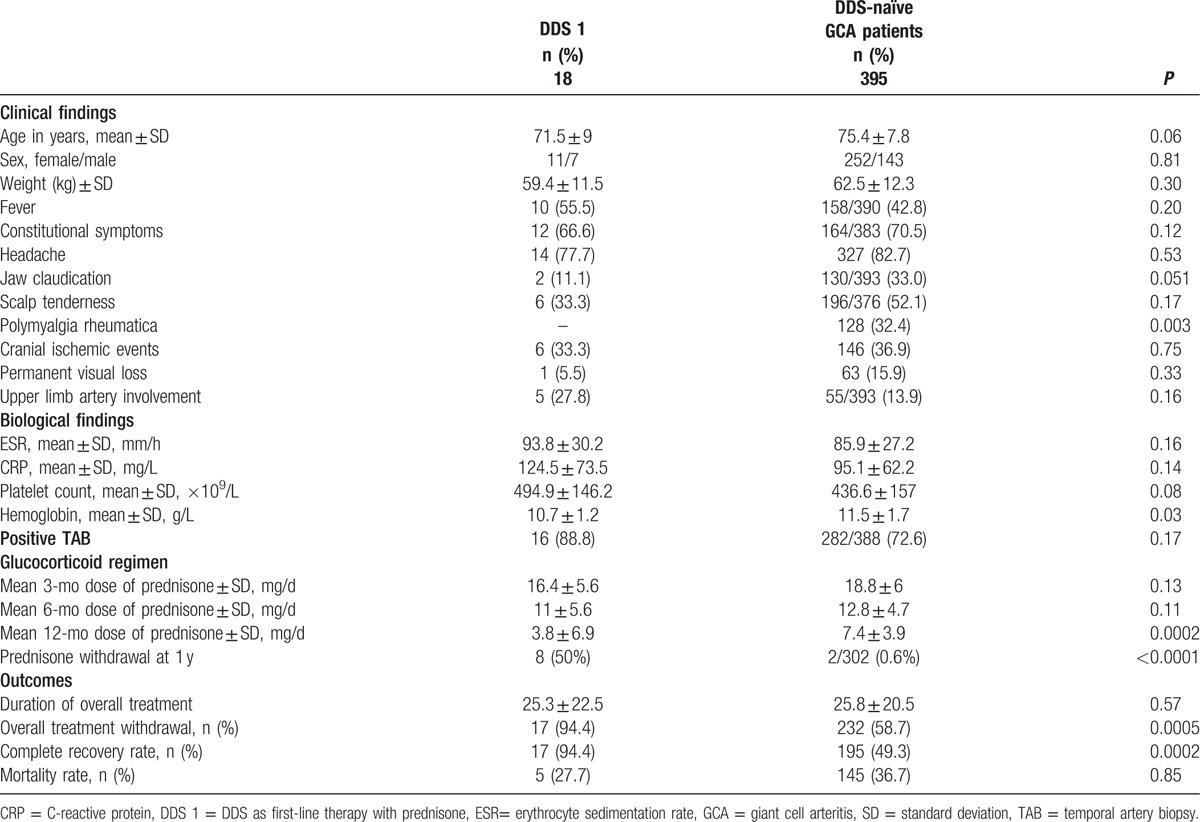
Comparison of first-line DDS-treated patients and DDS-naïve patients.

In all, 42 patients who received DDS (60%) recovered from GCA (mean time from treatment discontinuation 71 ± 70.1 months). The most frequent reasons for not achieving recovery were persistent or chronic GCA (17 patients), early death (4 patients), loss to follow-up (3 patients), and a too recent diagnosis (4 patients). In patients who recovered, GC treatment lasted for 35.4 ± 31.2 months overall, 21.4 ± 15.8 months in 17 DDS-1 patients, and 44.9 ± 35.5 months in 25 DDS-2 patients. The mean prednisone treatment duration was 16.4 ± 9.5 months in 14 DDS-1 patients who completed their DDS treatment, 13 of whom recovered from GCA; of these, 6 were treated for less than 1 year.

### 3.4 DDS-associated side effects and required clinical decisions

Side effects attributed to DDS were recorded in 44 of 69 (64%) assessable patients. The types and frequencies of DDS-related complications are shown in Table 4. These resulted in a successful reduction in the DDS dose by 25 mg/d in 11 patients (most often for significant hemolysis, excessive methemoglobinemia, or some clinical discomforts) and permanent cessation of DDS in 14 patients (20%) due to allergic skin rash in 7, agranulocytosis in 2, icteric hepatitis in 2, excessive hemolysis in 2, and poor digestive tolerance with fatigue in 1. Seven patients experienced excessive methemoglobinemia (4.1%–7%) during follow-up, not always within the first month, as expected, but in some cases up to 2 years after DDS initiation. Lowering of the DDS dose (e.g., to 25 mg/d) allowed DDS continuation in all patients. Of these patients, 5 had an initial daily DDS dose ≥75 mg. The highest methemoglobin level of 7% was recorded in an 82-year-old female diabetic patient who received 1.87 mg/kg/d DDS for 3 weeks.

**Table 4:**
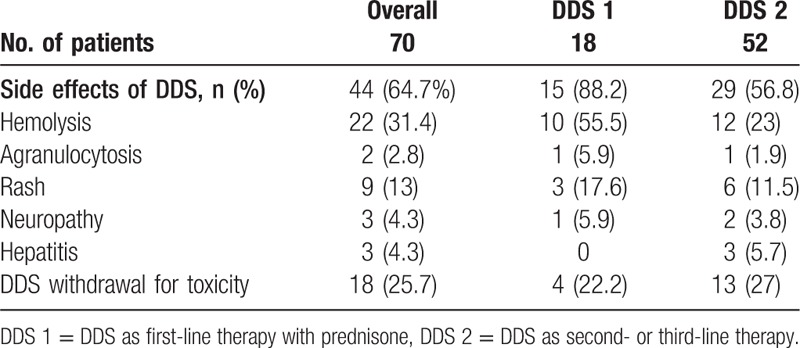
DDS-related adverse effects.

### 3.5 Use of other glucocorticoid-sparing drugs

Other GC-sparing drugs were given to 15 patients (21%). These patients belonged to the DDS-2 group, and 10 discontinued DDS within 4 months due to toxicity or weak efficacy. The DDS trial preceded other trials in 12 patients and was a third-line treatment in 3 patients. Regarding the patients from the inception cohort, 13 (21%) who took DDS received alternative treatments, compared with 11 (2.8%) of 394 patients from the same cohort who neither received DDS nor participated in another investigative therapeutic protocol. MTX was given to 10 patients, etanercept to 5, azathioprine to 3, TCZ to 3 patients, anakinra to 2, and mycophenolate mophetil to 1 patient (6 patients received at least 2 different treatments). The mean duration of GC treatment was 72.3 ± 53.6 months. Only 3 patients (20%) recovered from GCA during the mean 85 ± 50-month follow-up.

### 3.6 Death during treatment

The 70 patients were followed up for 88 ± 67.5 months. Eighteen of them died, 13 after having completed the treatment (Table 2). Five deaths occurred during GCA treatment, including our patients still taking DDS. The aforementioned 82-year-old female, who suffered a second GCA relapse complicated by bilateral ischemic visual loss, died on the day of her admission due to septic or hemorrhagic shock (autopsy not performed). The high blood methemoglobin level (7%) concurrently found might have been a precipitating factor in this patient. Three other fatalities occurred during DDS treatment but were clearly unrelated to treatment. One patient died from a massive stroke 4 months after GCA diagnosis and 3 months after the introduction of DDS; 1 patient died from pneumonia following mesenteric ischemia 8.5 months after diagnosis and 5.5 months after starting DDS; the last patient died from diastatic perforation of a colonic tumor 11 months after the GCA diagnosis and 8 months after DDS initiation. None of these 4 patients had experienced significant DDS-related side effects.

## 4 Discussion

Oral GCs are the mainstay of treatment for GCA, and the dose can be reduced to physiologic levels, that is, less than 7.5 mg/d within 12 months in most patients.^[8]^ However, many patients develop adverse side effects related to GCs, indicating that less-toxic therapeutic protocols are needed.^[8,29]^ Relapses, which occur in ∼50% of patients,^[2–5]^ require an increase in the GC dose, and some patients require long-term GC therapy at moderate-to-high doses, which increases the iatrogenic risk and may decrease survival.^[7,30]^ Such patients are defined as GC-dependent, although criteria for GC resistance or dependence and for disease remission have not been uniformly defined.^[31]^

Our results suggest the efficacy of DDS as a first-line treatment in addition to prednisone in GCA patients, with a greater reduction in the daily prednisone dose throughout the first year and a shorter duration of GC treatment compared with patients treated with prednisone alone. Although the majority of DDS-1 patients received adjunctive DDS without randomization, this group and the prednisone group appeared relatively well balanced in terms of clinical and laboratory features at diagnosis, which allowed comparison of the therapeutic results between the 2 groups. The most marked difference was a lower mean initial GC dose in the DDS-1 group, which is further evidence of DDS efficacy. The outcome of DDS-1 patients was consistent with that of patients included in the French multicenter protocol, in which the rate of recovery from GCA was higher and that of relapses after discontinuing therapy lower in the DDS-prednisone group compared with the prednisone-alone group, although the duration of steroid treatment was similar between the 2 groups.^[24]^ The present study shows that DDS provides a more sustained decrease in the GC dose, as demonstrated by a lower mean prednisone dose at 12 months, a higher proportion of patients achieving steroid withdrawal within the first year, cessation of prednisone treatment, and recovery from GCA (*P* < 0.001 for each variable). The difference in recovery rates between the DDS group and the control group may partly be explained by a longer mean follow-up in the former group (114 vs 63 months) and the unbalanced proportions of patients from the inception cohort who were followed up less than 2 years: 110 control patients (27.8%) versus 1 DDS-1 patient (5.6%).

Demonstrating a steroid-sparing effect of DDS as a second- or even third-line therapy in our patients with difficult-to-treat GCA is difficult, since a matched control group could not be defined owing to the retrospective design of the study. Reports on treatment of difficult-to-treat GCA with DDS are limited to an earlier report of 12 patients from our department and a further 4 cases.^[21,23]^Table 5 summarizes these previous studies. In the present study, at least 61% of the 38 assessable DDS-2 patients were able to significantly decrease their prednisone daily dose (from 23 ± 13.8 to 6.1 ± 6.0 mg/d; mean monthly decrease, 1.25 mg) while taking DDS, although this subset comprised a significant proportion of truly resistant patients. In most refractory cases, MTX and various biotherapies were also given but were not more effective than DDS, resulting in long-lasting GCA and no recovery in 80% of the cases.

**Table 5:**
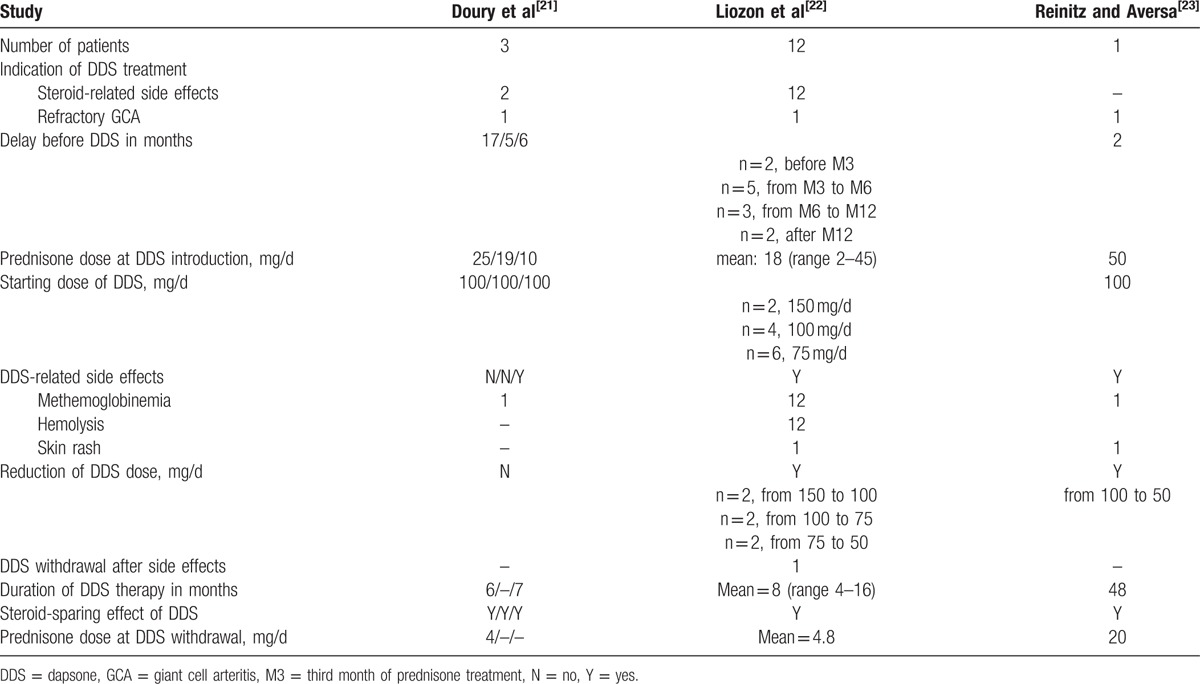
Open-label studies with DDS in refractory GCA patients.

A major issue with the use of DDS in GCA patients is its significant toxicity, which led to its permanent discontinuation in 14 patients (20%) in the present study. Major side effects were reported in 4 patients (6%), comprising agranulocytosis and icteric hepatitis in 2 patients each. Hospitalization was avoided in all 7 patients with reported allergic skin eruption, but DDS was discontinued permanently. Notably, these major events occurred within the first 3 months, significant side effects after this period being rare and usually controlled by a downward dose adjustment of 25 mg/d. The high blood methemoglobin level, a well known drawback of DDS,^[32,33]^ which is mediated by its oxidant properties,^[34]^ warrants particular attention. In the FNSIM study, the highest recorded methemoglobin level was 15%, which was successfully managed by reducing the daily DDS dose to 25 mg.^[24]^ Based on our experience, we recommend starting DDS at no more than 1 mg/kg/d, with a maximal dose of 100 mg/d, although upward adjustment can be performed as needed after 1 or 2 months in patients showing good drug tolerance. Furthermore, 3 patients exhibited a delayed increase in methemoglobin blood levels, indicating that at least monthly blood monitoring of methemoglobin levels throughout the course of DDS treatment is mandatory. Some patients develop significant anemia due to excessive hemolysis. In the present study, 22 patients (31%) had detectable hemolytic anemia, which was significant in 4 patients and led to DDS discontinuation in 1 patient. In the French multicenter protocol, maximum hemolysis occurred at the beginning of treatment and remained stable thereafter.^[24]^

To date, a direct comparison of DDS with other available steroid-sparing agents is lacking. In a prospective, randomized study, initial treatment of GCA with intravenous methylprednisolone pulses (1 g daily for 3 days) allowed for more rapid tapering of prednisone and had long-term benefits.^[35]^ However, no studies have focused on the efficacy of high-dose methylprednisolone in controlling relapsing or steroid-resistant GCA. Regarding the use of MTX in resistant GCA, prospective controlled trials have reported conflicting results,^[36–38]^ although a meta-analysis that recalculated the original data suggested the superiority of MTX after 24 months, since there were fewer relapses and lower GC doses in the MTX group.^[10]^ In 3 prospective studies, TNF-blockers lacked efficacy and cannot therefore be recommended in GCA.^[11,39,40]^ The steroid-sparing effect of DDS in difficult-to-treat GCA might be lower than that of cyclophosphamide, to which 88 of 103 patients (85%) responded.^[41]^ However, this figure was calculated by pooling results from retrospective series and case reports, which may involve significant biases.^[41]^ Finally, TCZ, a humanized monoclonal antibody targeting the IL-6 receptor, shows promise for GCA treatment.^[14]^ Indeed, several open-label studies using TCZ reported remission and maintenance thereof in refractory GCA patients.^[12,13,15,42,43]^ However, serious side effects including neutropenia, hepatitis, and infections were reported in these studies in up to 27%, and death occurred in 3 patients. Furthermore, data on the frequency of relapses after TCZ withdrawal are lacking in these studies. On the contrary, Régent et al provided insight into the curative effect of TCZ in refractory GCA. In this study, 6 of 34 (18%) patients experienced incomplete vasculitis remission during TCZ treatment, 1 patient died, and 3 patients had to stop TCZ treatment because of severe adverse events. Of 23 patients who were able to stop treatment, 8 suffered recurrence after a mean of 3.5 ± 1.3 months.^[44]^ Unizony et al reported a patient who died postoperatively of myocardial infarction 6 months after TCZ withdrawal. Autopsy revealed persistent vasculitis of large- and medium-sized arteries.^[15]^ Taken together, these data indicate that TCZ is effective in suppressing the vascular and systemic inflammation of GCA but might have, at least in some patients, a transient effect without ensuring true recovery. Furthermore, TCZ is far more costly than DDS, is suitable only on an inpatient basis, and has several serious side effects.

In conclusion, our findings suggest DDS to be a potent GC-sparing agent in patients with GCA, which should be evaluated in prospective studies. However, because of its toxicity, DDS use should be restricted to refractory cases and patients who experience unacceptable GC-induced complications. After initiation of DDS treatment, close clinical and laboratory (complete blood counts, blood methemoglobin level, and hepatic tests) monitoring is needed, at weekly intervals within the first 3 months and monthly thereafter.
